# Effect of palbociclib plus endocrine therapy on time to chemotherapy across subgroups of patients with hormone receptor‒positive/human epidermal growth factor receptor 2‒negative advanced breast cancer: Post hoc analyses from PALOMA-2 and PALOMA-3

**DOI:** 10.1016/j.breast.2022.11.005

**Published:** 2022-11-15

**Authors:** Hope S. Rugo, Seock-Ah Im, Anil A. Joy, Yaroslav Shparyk, Janice M. Walshe, Bethany Sleckman, Sherene Loi, Kathy Puyana Theall, Sindy Kim, Xin Huang, Eustratios Bananis, Reshma Mahtani, Richard S. Finn, Véronique Diéras

**Affiliations:** a University of California, San Francisco, Helen Diller Family Comprehensive Cancer Center, Department of Medicine (Hematology/Oncology), 1825 4th Street, 3rd Floor, Box 1710, San Francisco, CA, 94158, USA; b Seoul National University Hospital, Cancer Research Institute, Seoul National University College of Medicine, Seoul National University, 101 Daehak-ro, Jonro-gu, Seoul 03080, Republic of Korea; c Cross Cancer Institute, University of Alberta, 11560 University Ave NW, Edmonton, AB T6G1Z2, Canada; d Lviv State Oncologic Regional Treatment and Diagnostic Center, Lviv, Ukraine; e Cancer Trials Ireland, St Vincent's University Hospital, Elm Park, Dublin 4, Ireland; f Mercy Hospital St. Louis, 607 S New Ballas Road, Suite 3300, St. Louis, MO, 63141, USA; g Peter MacCallum Cancer Centre, University of Melbourne, Australia; h Pfizer Oncology, 300 Technology Square, Cambridge, MA 02139, USA; i Pfizer Inc, 10555 Science Center Dr, San Diego, CA 92121, USA; j Pfizer Inc, 235 E 42nd St, New York, NY, 10017, USA; k Miami Cancer Institute, Baptist Health South Florida, Member, Memorial Sloan Kettering Cancer Alliance, 1228 South Pine Island Road, Plantation, FL, 33324, USA; l David Geffen School of Medicine, 2825 Santa Monica Blvd, Suite 200, Santa Monica, CA, 90404, USA; m Unicancer Centre Eugène Marquis, Avenue de la Bataille Flandres-Dunkerque, CS 44229, 35042, Rennes Cedex, France

**Keywords:** Advanced breast cancer, Chemotherapy, Palbociclib, Progression-free survival, Safety

## Abstract

**Background:**

Previous analyses from the PALOMA-2 and PALOMA-3 studies showed that palbociclib (PAL) plus endocrine therapy (ET) prolongs time to first subsequent chemotherapy (TTC) versus placebo (PBO) plus ET in the overall population of patients with hormone receptor‒positive/human epidermal growth factor receptor 2‒negative (HR+/HER2−) advanced breast cancer (ABC). Here, we evaluated TTC in relevant patient subgroups.

**Methods:**

These post hoc analyses evaluated TTC by subgroup using data from 2 randomized, phase 3 studies of women with HR+/HER2− ABC. In PALOMA-2, postmenopausal patients previously untreated for ABC were randomized 2:1 to receive PAL (125 mg/day, 3/1-week schedule) plus letrozole (LET; 2.5 mg/day; n = 444) or PBO plus LET (n = 222). In PALOMA-3, premenopausal or postmenopausal patients whose disease had progressed after prior ET were randomized 2:1 to receive PAL (125 mg/day, 3/1-week schedule) plus fulvestrant (FUL; 500 mg; n = 347) or PBO plus FUL (n = 174).

**Results:**

First subsequent chemotherapy was received by 35.5% and 56.2% in PALOMA-2 and PALOMA-3 after progression on palbociclib plus ET or placebo plus ET. Across all subgroups analyzed, the median progression-free survival (PFS) was longer in the PAL plus ET arm than the PBO plus ET arm. TTC was longer with PAL plus ET versus PBO plus ET across the same patient subgroups in both studies.

**Conclusions:**

Across all subgroups, PAL plus ET versus PBO plus ET had longer median PFS and resulted in prolonged TTC in both the PALOMA-2 and PALOMA-3 studies.

Pfizer Inc (NCT01740427, NCT01942135).

## Highlights

• Palbociclib + endocrine therapy prolongs time to chemotherapy in HR+/HER2– cancer.
• We analyzed palbociclib efficacy data from 2 clinical trials across subgroups.
• Patient subgroups were based on demographics and baseline disease characteristics.
• Palbociclib increased progression-free survival versus placebo across all subgroups.
• Palbociclib increased time to chemotherapy versus placebo across all subgroups.

## 1 Introduction

Cyclin-dependent kinases (CDKs) belong to the serine-threonine kinases and are activated by D-type cyclins [1,2]. CDKs, particularly CDK4 and CDK6 (CDK4/6), regulate cell cycle progression from the G0 or G1 phase into the S phase by phosphorylating the tumor suppressor gene retinoblastoma and other related proteins like p107 and p130 [1,3]. Palbociclib (Ibrance, Pfizer) is a small molecule inhibitor of CDK4/6 with a high selectivity profile toward CDK4/6 over other CDKs [4,5].

For women with hormone receptor‒positive/human epidermal growth factor receptor 2‒negative (HR+/HER2−) advanced breast cancer (ABC), CDK4/6 inhibitors in combination with endocrine therapy (ET) have become the standard of care [6,7]. The first-in-class CDK4/6 inhibitor palbociclib in combination with ET is approved to treat patients with HR+/HER− ABC based on the demonstration of prolonged progression-free survival (PFS) and an acceptable safety profile in phase 3 trials in patients with HR+/HER− ABC who were previously untreated (PALOMA-2; NCT01740427) and in patients who had relapsed or progressed during prior ET and could have received 1 prior line of chemotherapy for ABC (PALOMA-3; NCT01942135) [5,8]. In individual analyses of the phase 3 PALOMA-2 and PALOMA-3 trials, time to subsequent chemotherapy (TTC) after discontinuation of study treatment was prolonged in patients in the palbociclib arm compared with the placebo arm [9,10]. In PALOMA-2, the median TTC was 40.4 [95% CI, 34.7–47.3] months versus 29.9 [95% CI, 25.6–35.1] months for patients in the palbociclib plus ET versus placebo plus ET arm, respectively (hazard ratio [HR], 0.74 [95% CI, 0.59–0.92]) [9]. In PALOMA-3, the TTC was 17.6 [95% CI, 15.2–19.7] months versus 8.8 [95% CI, 7.3–12.7] months in the palbociclib plus ET versus placebo plus ET arm, respectively (HR, 0.58 [95% CI, 0.47–0.73]; *P* < 0.001) [10]. In these post hoc analyses, we evaluated TTC in subgroups of patients with HR+/HER2− ABC from each of the PALOMA-2 and PALOMA-3 trials.

## 2 Patients and methods

### 2.1 Study design and patients

PALOMA-2 and PALOMA-3 were phase 3, double-blind, randomized, placebo-controlled studies of palbociclib plus ET in patients with HR+/HER2− ABC [5,8]. In PALOMA-2, postmenopausal women (N = 666) with previously untreated, estrogen receptor‒positive/HER2– ABC were randomized 2:1 to receive palbociclib (125 mg daily in 4-week cycles on a 3/1 schedule [3 weeks on/1 week off]) or placebo; patients in both arms received letrozole (LET; 2.5 mg daily; continuous treatment) [8].

In PALOMA-3, women (N = 521) of any menopausal status with HR+/HER2− ABC whose disease had progressed after any number of lines of prior ET and who received up to 1 prior chemotherapy regimen for ABC were randomized 2:1 to receive palbociclib (125 mg daily, 3/1 schedule) plus fulvestrant (FUL; 500 mg every 14 days for the first 3 injections and then every 28 days) or placebo plus FUL [5]. Patients who were premenopausal or perimenopausal received concurrent ovarian suppression with goserelin [5]. Approximately 34% of patients who participated in PALOMA-3 had received prior chemotherapy for their advanced disease at baseline [10].

### 2.2 Data analysis

Numbers and percentages of patients who received first subsequent chemotherapy after discontinuing study treatment were calculated by treatment group in the PALOMA-2 and PALOMA-3 intent-to-treat (ITT) populations and in subgroups of patients according to demographics and baseline disease characteristics. Separate analyses were performed for the individual studies.

The Kaplan-Meier method was used to estimate median TTC and PFS in the ITT population and in patient subgroups by treatment arm along with corresponding 95% CIs based on the Brookmeyer and Crowley method [9]. Unstratified HRs for PFS and TTC were estimated using the Cox proportional hazards model with associated 95% CIs. HR < 1 indicated a reduction in the hazard rate in favor of palbociclib.

## 3 Results

### 3.1 PALOMA-2

In PALOMA-2, at data cut-off (May 31, 2017), a total of 444 women had received palbociclib plus LET and 222 women had received placebo plus LET. After discontinuation of study treatment, 35.5% of patients had received first subsequent chemotherapy (36.6% in palbociclib plus LET group and 34% in placebo plus LET group). Demographics and baseline disease characteristics for patients with versus without first subsequent chemotherapy are presented in Table 1. Patients in both the palbociclib plus LET and placebo plus LET arms were more likely to receive versus not receive first subsequent chemotherapy after discontinuation of study treatment if they had visceral disease, a disease-free interval (DFI) of ≤ 12 months, or received prior adjuvant or neoadjuvant systemic or hormonal therapy or chemotherapy.

**Table 1:** Demographics and baseline disease characteristics for patients who did and did not receive first subsequent CT in PALOMA-2 by treatment arm.

	Without First Subsequent CT		With First Subsequent CT	
	PAL + LET	PBO + LET	PAL + LET	PBO + LET
	(n = 361)	(n = 171)	(n = 83)	(n = 51)
Age, median, y	63.0	61.0	59.0	61.0
< 65, n (%)	206 (57.1)	108 (63.2)	57 (68.7)	33 (64.7)
≥ 65, n (%)	155 (42.9)	63 (36.8)	26 (31.3)	18 (35.3)
Race,*n (%)				
White	275 (76.2)	130 (76.0)	69 (83.1)	42 (82.4)
Black	6 (1.7)	3 (1.8)	2 (2.4)	0
Asian	55 (15.2)	25 (14.6)	10 (12.0)	5 (9.8)
Other	25 (6.9)	13 (7.6)	2 (2.4)	4 (7.8)
Weight, median, kg	67.3	66.7	71.2	67.0
Disease site, n (%)				
Visceral	167 (46.3)	76 (44.4)	47 (56.6)	34 (66.7)
Nonvisceral	194 (53.7)	95 (55.6)	36 (43.4)	17 (33.3)
Disease-free completion of prior (neo)adjuvant therapy, n (%)				
De novo metastatic	141 (39.1)	65 (38.0)	26 (31.3)	16 (31.4)
DFI ≤ 12 mo	69 (19.1)	33 (19.3)	29 (34.9)	15 (29.4)
DFI > 12 mo	151 (41.8)	73 (42.7)	28 (33.7)	20 (39.2)
Nature of prior (neo)adjuvant anticancer therapy, n (%)				
Prior systemic therapy				
No	141 (39.1)	65 (38.0)	26 (31.3)	16 (31.4)
Yes	220 (60.9)	106 (62.0)	57 (68.7)	35 (68.6)
Prior CT for primary diagnosis, n (%)				
No	194 (53.7)	92 (53.8)	37 (44.6)	21 (41.2)
Yes	167 (46.3)	79 (46.2)	46 (55.4)	30 (58.8)
Neoadjuvant	39 (10.8)	23 (13.5)	15 (18.1)	9 (17.6)
Adjuvant	143 (39.6)	64 (37.4)	37 (44.6)	25 (49.0)
Prior hormonal therapy for primary diagnosis, n (%)				
No	163 (45.2)	77 (45.0)	31 (37.3)	19 (37.3)
Yes	198 (54.8)	94 (55.0)	52 (62.7)	32 (62.7)
Prior hormonal therapies, n (%)				
1	128 (35.5)	62 (36.3)	30 (36.1)	25 (49.0)
> 1	70 (19.4)	32 (18.7)	22 (26.5)	7 (13.7)

CT = chemotherapy; DFI = disease-free interval; LET = letrozole; PAL = palbociclib; PBO = placebo.

*Other includes not reported/missing patients.

**Fig. 1:**
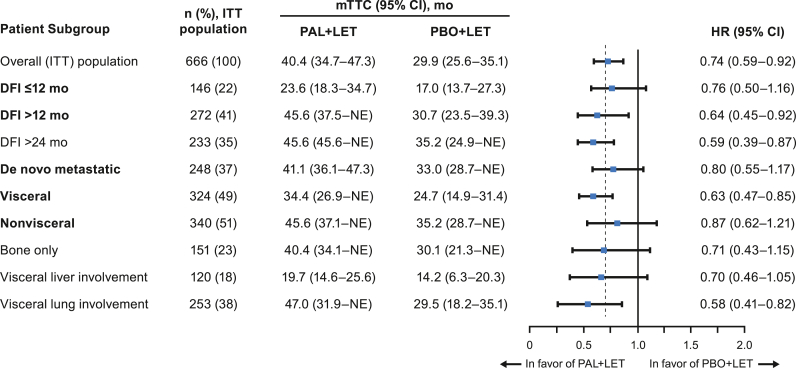
Forest plot of TTC by treatment arm in PALOMA-2, overall and across patient subgroups (ITT population). DFI = disease-free interval; HR = hazard ratio; ITT = intent to treat; LET = letrozole; (m) TTC=(median) time to first subsequent chemotherapy; mo = months; NE = not estimable; PAL = palbociclib; PBO = placebo. Items in bold in the Patient Subgroup column represent stratification factors from PALOMA-2.

**Fig. 2:**
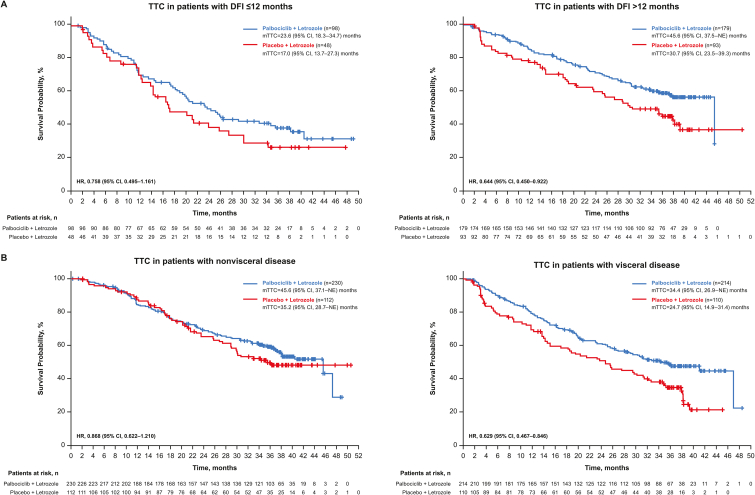
TTC in subgroups of patients from PALOMA-2 with (A) DFI ≤ 12 months and > 12 months and (B) nonvisceral and visceral disease (ITT population). DFI = disease-free interval; HR = hazard ratio; ITT = intent to treat; (m)TTC=(median) time to first subsequent chemotherapy; NE = not estimable.

The median PFS was longer in the palbociclib plus LET arm compared with the placebo plus LET arm across the patient subgroups from PALOMA-2 (Table 2), as previously reported [9,11]. Across all subgroups included in this analysis, the TTC was longer with palbociclib plus LET compared with placebo plus LET (Fig. 1). Patients with DFI ≤ 12 months had a median TTC of 23.6 (95% CI, 18.3–34.7) months in the palbociclib plus LET arm versus 17.0 (95% CI, 13.7–27.3) months in the placebo plus LET arm (HR, 0.76 [95% CI, 0.50–1.16]); for patients with DFI > 12 months, the median TTC was 45.6 (95% CI, 37.5–Not Estimable [NE]) months versus 30.7 (95% CI, 23.5–39.3) months, respectively (HR, 0.64 [95% CI, 0.45–0.92]; Fig. 2A). Similar results were observed among patients with visceral and nonvisceral disease. Those with nonvisceral disease had a median TTC of 45.6 (95% CI, 37.1–NE) months versus 35.2 (95% CI, 28.7–NE) months in the palbociclib plus LET versus placebo plus LET arms, respectively (HR, 0.87 [95% CI, 0.62–1.21]); for patients with visceral disease, the median TTC was 34.4 (95% CI, 26.9–NE) months versus 24.7 (95% CI, 14.9–31.4) months, respectively (HR, 0.63 [95% CI, 0.47–0.85]; Fig. 2B).

**Table 2:** PFS in patients from PALOMA-2 by subgroup and treatment arm.

Patient Subgroup	ITT Population, n (%)	mPFS (95% CI), mo		HR (95% CI)
		PAL + LET	PBO + LET	
Overall (ITT) population	666 (100)	27.6 (22.4–30.3)	14.5 (12.3–17.1)	0.56 (0.46–0.69)
**DFI** ≤ **12 mo**	146 (22)	16.6 (13.9–24.2)	11.0 (5.6–12.9)	0.48 (0.32–0.72)
**DFI** > **12 mo**	272 (41)	30.3 (24.8‒NE)	13.8 (8.8–18.2)	0.55 (0.40–0.76)
DFI > 24 mo	233 (35)	38.5 (27.5‒NE)	16.6 (13.7–23.5)	0.52 (0.36–0.75)
**De novo metastatic**	248 (37)	27.9 (22.1–33.4)	22.0 (13.9–27.4)	0.61 (0.44–0.85)
**Visceral**	324 (49)	19.3 (16.4–24.2)	12.3 (8.4–16.4)	0.62 (0.47–0.81)
**Nonvisceral**	340 (51)	35.9 (27.7‒NE)	17.0 (13.8–24.8)	0.50 (0.37–0.67)
Bone only	151 (23)	36.2 (27.6‒NE)	11.2 (8.2–22.0)	0.41 (0.26–0.63)
Visceral liver involvement	120 (18)	13.7 (10.9–16.6)	8.4 (5.5–12.9)	0.62 (0.41–0.94)
Visceral lung involvement	253 (38)	23.2 (17.0–27.8)	12.9 (8.1–16.6)	0.58 (0.42–0.80)

DFI = disease-free interval; HR = hazard ratio; ITT = intent to treat; LET = letrozole; (m)PFS=(median) progression-free survival; NE = not estimable; PAL = palbociclib; PBO = placebo. Items in bold in the Patient Subgroup column represent stratification factors from PALOMA-2.

After a median follow-up of 38 months, approximately 55% of patients in the palbociclib plus LET arm and 73% of patients in the placebo plus LET arm received follow-up post-trial systemic anti-cancer therapy (Supplemental Table 1). Antihormonal therapy (FUL and exemestane) was used in about 60% of patients in both groups for first subsequent therapy and in 36% and 49% of patients, respectively, in the palbociclib plus LET arm and the placebo plus LET arm for second subsequent therapy (Supplemental Table 2). Paclitaxel and capecitabine were the most commonly used chemotherapy agents for both first and second subsequent therapy.

### 3.2 PALOMA-3

In PALOMA-3, at data cut-off (August 17, 2020), a total of 347 women had received palbociclib plus FUL and 174 women had received placebo plus FUL. After discontinuation of study treatment, 56.6% of patients had received first subsequent chemotherapy (53.6% in palbociclib plus FUL group and 61.1% in placebo plus FUL group). Demographics and baseline disease characteristics for patients with versus without first subsequent chemotherapy after study drug discontinuation are presented in Table 3. A higher percentage of younger patients (< 65 years of age) received versus did not receive first subsequent chemotherapy after discontinuation in the palbociclib plus FUL arm (81.1% vs 71.1%). In the placebo plus FUL arm, a higher percentage of patients with premenopausal or perimenopausal status received versus did not receive first subsequent chemotherapy after treatment discontinuation (29.5% vs 11.6%). Among those with visceral disease, a higher percentage received versus did not receive first subsequent chemotherapy after discontinuation of either palbociclib plus FUL (63.6% vs 53.4%) or placebo plus FUL (69.3% vs 50.0%). A higher percentage of patients who had prior chemotherapy in the metastatic setting received versus did not receive first subsequent chemotherapy in both the palbociclib plus FUL arm (35.7% vs 30.4%) and placebo plus FUL arm (43.2% vs 30.2%). Similar results were observed among those who received prior hormonal therapies.

**Table 3:** Select patient demographics and baseline disease characteristics for patients who did or did not receive first subsequent CT in PALOMA-3 by treatment arm.

	Without First Subsequent CT		With First Subsequent CT	
	PAL + FUL	PBO + FUL	PAL + FUL	PBO + FUL
	(n = 204)	(n = 86)	(n = 143)	(n = 88)
Age, median, y	58.0	60.0	56.0	54.0
< 65 y, n (%)	145 (71.1)	64 (74.4)	116 (81.1)	67 (76.1)
≥ 65 y, n (%)	59 (28.9)	22 (25.6)	27 (18.9)	21 (23.9)
Race,*n (%)				
White	150 (73.5)	68 (79.1)	102 (71.3)	65 (73.9)
Black	8 (3.9)	4 (4.7)	4 (2.8)	4 (4.5)
Asian	40 (19.6)	14 (16.3)	34 (23.8)	17 (19.3)
Other	6 (2.9)	0	2 (1.4)	1 (1.1)
Unspecified	0	0	1 (0.7)	1 (1.1)
Weight, median, kg	67.1	71.2	69.0	68.4
Menopausal status, n (%)				
Premenopausal/perimenopausal	39 (19.1)	10 (11.6)	33 (23.1)	26 (29.5)
Postmenopausal	165 (80.9)	76 (88.4)	110 (76.9)	62 (70.5)
Disease site, n (%)				
Visceral	109 (53.4)	43 (50.0)	91 (63.6)	61 (69.3)
Nonvisceral	95 (46.6)	43 (50.0)	52 (36.4)	27 (30.7)
Sensitivity to prior ET, n (%)				
Yes	162 (79.4)	65 (75.6)	111 (77.6)	68 (77.3)
No	42 (20.6)	21 (24.4)	32 (22.4)	20 (22.7)
Prior CT for primary diagnosis, n (%)				
No	59 (28.9)	17 (19.8)	35 (24.5)	19 (21.6)
Yes	145 (71.1)	69 (80.2)	108 (75.5)	69 (78.4)
Neoadjuvant	41 (20.1)	15 (17.4)	26 (18.2)	18 (20.5)
Adjuvant	87 (42.6)	48 (55.8)	64 (44.8)	41 (46.6)
Metastatic	62 (30.4)	26 (30.2)	51 (35.7)	38 (43.2)
Missing	0	1 (1.2)	1 (0.7)	0
Prior hormonal therapy, n (%)				
1	78 (38.2)	37 (43.0)	48 (33.6)	34 (38.6)
> 1	126 (61.8)	49 (57.0)	95 (66.4)	54 (61.4)
Metastatic	151 (74.0)	64 (74.4)	108 (75.5)	64 (72.7)
Prior lines of therapy for ABC, n (%)				
0/1	135 (66.2)	61 (70.9)	80 (55.9)	63 (71.6)
≥ 2	69 (33.8)	25 (29.1)	63 (44.1)	25 (28.4)

ABC = advanced breast cancer; CT = chemotherapy; ET = endocrine therapy; FUL = fulvestrant; PAL = palbociclib; PBO = placebo.

*Other includes not reported/missing patients.

**Fig. 3:**
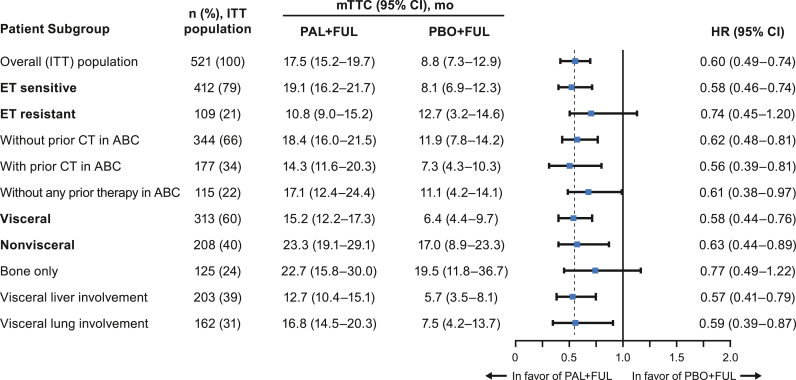
Forest plot of TTC by treatment arm in PALOMA-3, overall and across patient subgroups (ITT population). ABC = advanced breast cancer; CT = chemotherapy; ET = endocrine therapy; FUL = fulvestrant; HR = hazard ratio; ITT = intent to treat; mo = months; (m)TTC=(median) time to first subsequent chemotherapy; PAL = palbociclib; PBO = placebo. Items in bold in the Patient Subgroup column represent stratification factors from PALOMA-3.

**Fig. 4:**
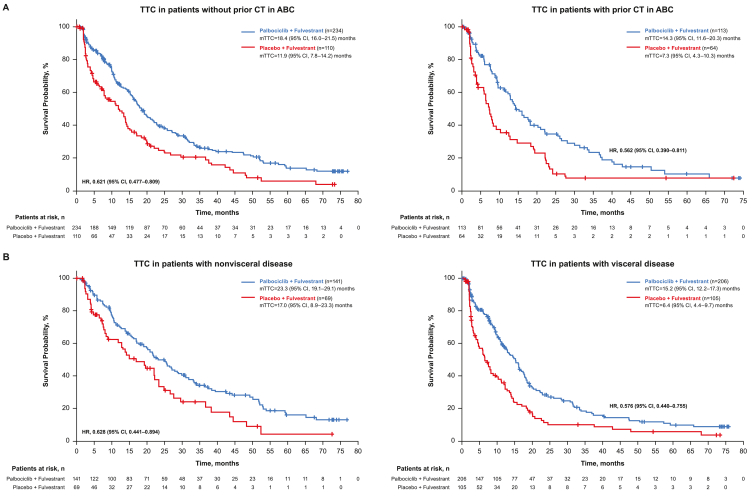
TTC in subgroups of patients from PALOMA-3 (A) with and without prior chemotherapy in ABC and (B) with nonvisceral and visceral disease (ITT population). ABC = advanced breast cancer; CT = chemotherapy; HR = hazard ratio; ITT = intent to treat; (m)TTC=(median) time to first subsequent chemotherapy.

The median PFS was longer in the palbociclib plus FUL arm compared with the placebo plus FUL arm across the patient subgroups who had disease progression during prior ET from PALOMA-3 (Table 4), as previously reported [[11], [12], [13]]. Across all subgroups from PALOMA-3 included in this analysis, TTC was longer with palbociclib plus FUL compared with placebo plus FUL (Fig. 3). Patients without prior chemotherapy in the ABC setting had a median TTC of 18.4 (95% CI, 16.0–21.5) months versus 11.9 (95% CI, 7.8–14.2) months in the palbociclib plus FUL arm versus the placebo plus FUL arm, respectively (HR, 0.62 [95% CI, 0.48–0.81]); for patients with prior chemotherapy in the ABC setting, the median TTC was 14.3 (95% CI, 11.6–20.3) months versus 7.3 (95% CI, 4.3–10.3) months, respectively (HR, 0.56 [95% CI, 0.39–0.81]; Fig. 4A). Patients with nonvisceral disease had a median TTC of 23.3 (95% CI, 19.1–29.1) months versus 17.0 (95% CI, 8.9–23.3) months in the palbociclib plus FUL arm versus the placebo plus FUL arm, respectively (HR, 0.63 [95% CI, 0.44–0.89]); for patients with visceral disease, the median TTC was 15.2 (95% CI, 12.2–17.3) months versus 6.4 (95% CI, 4.4–9.7) months, respectively (HR, 0.58 [95% CI, 0.44–0.76]; Fig. 4B).

**Table 4:** PFS in patients from PALOMA-3 by subgroup and treatment arm.

Patient Subgroup	ITT Population, n (%)	mPFS (95% CI), mo		HR (95% CI)
		PAL + FUL	PBO + FUL	
Overall (ITT) population	521 (100)	11.2 (9.5–12.9)	4.6 (3.5–5.6)	0.50 (0.40–0.62)
**ET sensitive**	412 (79)	12.0 (11.1–13.9)	4.2 (3.5–5.6)	0.46 (0.36–0.59)
**ET resistant**	109 (21)	7.4 (5.5–11.1)	5.1 (1.9–7.4)	0.69 (0.43–1.09)
Without prior CT in ABC	344 (66)	12.9 (11.0–15.0)	5.5 (3.6–7.6)	0.49 (0.37–0.65)
With prior CT in ABC	177 (34)	9.5 (7.3–11.3)	3.5 (1.9–5.4)	0.54 (0.37–0.77)
Without any prior therapy in ABC	115 (22)	11.0 (7.3–13.2)	5.1 (2.1–9.2)	0.59 (0.37–0.93)
**Visceral**	313 (60)	9.2 (7.5–11.1)	3.5 (2.0–5.1)	0.50 (0.38–0.65)
**Nonvisceral**	208 (40)	16.6 (13.2‒NE)	5.6 (4.6–10.9)	0.48 (0.33–0.71)
Bone only	125 (24)	14.3 (11.2‒NE)	9.2 (4.8–20.0)	0.63 (0.38–1.06)
Visceral liver involvement	203 (39)	7.5 (5.6–9.2)	2.4 (1.9–3.6)	0.49 (0.36–0.68)
Visceral lung involvement	162 (31)	11.1 (9.2–12.0)	3.7 (2.1–7.2)	0.45 (0.31–0.67)

ABC = advanced breast cancer; CT = chemotherapy; ET = endocrine therapy; FUL = fulvestrant; HR = hazard ratio; ITT = intent to treat; (m) PFS=(median) progression-free survival; NE = not estimable; PAL = palbociclib; PBO = placebo. Items in bold in the Patient Subgroup column represent stratification factors from PALOMA-3.

After a median follow-up of 73.3 months, approximately 77% of patients in the palbociclib plus FUL arm and 83% of patients in the placebo plus FUL arm received follow-up post-trial systemic anti-cancer therapy (Supplemental Table 1). Chemotherapy was the most common subsequent therapy in the palbociclib and control groups and increased across first (54%–61%), second (66%–72%), and third (90%–92%) subsequent therapies. Antihormonal therapy (exemestane and FUL) were commonly used in 20%–43% of patients across first, second, and third subsequent therapies (Supplemental Table 3).

## 4 Discussion

In the PALOMA-2 and PALOMA-3 studies, after treatment discontinuation, 35.5% and 56.2% of patients had received first subsequent chemotherapy, respectively; across all subgroups, patients treated with palbociclib plus ET had longer median PFS that resulted in prolonged TTC compared with those patients treated with placebo plus ET. Similar benefits were also observed with other CDK4/6 inhibitors in the MONARCH-3 and MONALEESA-2 studies. In MONARCH-3, an exploratory analysis showed that abemaciclib in combination with an aromatase inhibitor delayed the initiation of first chemotherapy (stratified HR, 0.513 [95% CI, 0.380–0.691]) in postmenopausal women with HR+/HER2−, locoregionally recurrent or metastatic breast cancer [14]. In this study, patients who died (abemaciclib arm, 14.3%; placebo arm, 7.9%) before the initiation of chemotherapy were excluded [14]. In MONALEESA-2, postmenopausal patients with HR+/HER2− ABC who were treated with ribociclib plus ET had an approximately 1-year delay to first chemotherapy compared with those who received placebo plus ET (median, 50.6 months vs 38.9 months; HR, 0.74 [95% CI, 0.61–0.91]) [15].

Across all subgroups analyzed in PALOMA-2 and PALOMA-3, the median PFS was longer in the palbociclib plus ET arm compared with the placebo plus ET arm. Furthermore, TTC was longer with palbociclib plus ET compared with placebo plus ET, including in patients with visceral disease, prior chemotherapy in ABC, and DFI ≤ 12 months. Regardless of treatment, patients with poor prognosis (DFI ≤ 12 months, ET resistance, visceral disease, and prior chemotherapy for ABC) had shorter median PFS and TTC compared with patients with better prognosis (DFI > 12 months, de novo, ET sensitivity, nonvisceral/bone-only, and no prior chemotherapy for ABC). These finding were also observed when examining the demographics and characteristics of patients who went on to receive first subsequent chemotherapy compared with those who did not. Patients who went on to receive first subsequent chemotherapy were more likely to have DFI ≤ 12 months, liver or visceral metastases, and prior chemotherapy in the neoadjuvant, adjuvant, or ABC setting. As expected, patients who received palbociclib plus ET in the first-line setting (ie, no prior therapy for ABC) had a longer median PFS and TTC than patients who received palbociclib plus ET after progressing on ET.

These data from PALOMA-2 and PALOMA-3 should be considered in light of potential study limitations, including the exploratory and post hoc nature of the studies. Considering the small number of patients in some of the subgroups, the data should be interpreted with caution. Taken together, these findings in combination with the current body of literature regarding PFS and TTC benefits with CDK4/6 inhibitors suggest that patients receive greater clinical benefit from palbociclib plus ET compared with ET monotherapy for the treatment of HR+/HER2− ABC.

## Data sharing statement

Upon request, and subject to review, Pfizer will provide the data that support the findings of this study. Subject to certain criteria, conditions and exceptions, Pfizer may also provide access to the related individual de-identified participant data. See https://www.pfizer.com/science/clinical-trials/trial-data-and-results for more information.

## Role of the funding source

This study was sponsored by 10.13039/100004319Pfizer Inc. The sponsor was involved in study design and in the collection, analysis and interpretation of data. Eustratios Bananis, Xin Huang, Kathy Puyana Theall and Sindy Kim, who are authors of this manuscript, are employees of and stockholders in Pfizer Inc, and therefore were involved in the writing of the manuscript and in the decision to submit the manuscript for publication.

## Declaration of competing interest

**Hope S. Rugo** reports sponsored research to her institution from 10.13039/100004319Pfizer Inc, 10.13039/100004334Merck, 10.13039/100004336Novartis, Eli Lilly, 10.13039/100004337Roche, 10.13039/501100002973Daiichi-Sankyo, 10.13039/100010293Seattle Genetics, Macrogenics, Sermonix, 10.13039/100001003Boehringer Ingelheim, Polyphor, 10.13039/100004325AstraZeneca, Ayala, and 10.13039/100005564Gilead and honoraria from PUMA, 10.13039/100004358Samsung, and 10.13039/100016259Mylan.

**Seock-AhIm** has received research funding from 10.13039/100004325AstraZeneca, 10.13039/501100004470Daewoong Pharm, 10.13039/501100003769Eisai, 10.13039/100004319Pfizer, and 10.13039/100004337Roche and reports consulting fees for AstraZeneca, Daiichi-Sankyo, Eli Lilly, GSK, Hanmi, Idience, MSD, Novartis, Pfizer Inc, and Roche.

**Sherene Loi** has received research funding to her institution from 10.13039/100004336Novartis, Bristol Myers Squibb, 10.13039/100004334Merck, Puma Biotechnology, Eli Lilly, Nektar Therapeutics, 10.13039/100004325AstraZeneca, 10.13039/100004328Roche-Genentech, and 10.13039/100010293Seattle Genetics, consulting fees paid to her institution from Aduro Biotech, Novartis, GlaxoSmithKline, Roche-Genentech, AstraZeneca, Silverback Therapeutics, G1 Therapeutics, PUMA Biotechnologies, Pfizer Inc, Gilead Therapeutics, Seattle Genetics, Tallac Therapeutics, and Bristol Meyers Squibb, is a Scientific Advisory Board Member of Akamara Therapeutics, has acted as a consultant (not compensated) to Seattle Genetics, Novartis, Bristol Meyers Squibb, Merck, AstraZeneca, and Roche-Genentech, and is supported by the 10.13039/501100001026National Breast Cancer Foundation of Australia Endowed Chair and the 10.13039/100001006Breast Cancer Research Foundation, New York.

**Anil A Joy** reports consulting fees from Pfizer, Novartis, Roche, Eli Lilly, AstraZeneca, Bristol Myers Squibb, Boehringer Ingelheim, Abbvie, Amgen, Genomic Health, Puma, and Teva, is an Advisory Board member of Pfizer, and is a part of the National Study Chair NCIC/CCTG MA 38.

**Eustratios Bananis, Xin Huang, Kathy Puyana Theall,** and **Sindy Kim** are employees of and stockholders in Pfizer Inc.

**Richard S. Finn** reports consulting fees/honoraria from 10.13039/100004319Pfizer Inc and research grant/funding from 10.13039/100004319Pfizer Inc, Eli Lilly, and 10.13039/100004336Novartis.

**Yaroslav Shparyk** has no conflict of interest.

**Janice M. Walshe** reports consulting fees (eg, advisory boards) from Roche, Genomic Health, and Pfizer, and reports fees for non-CME Services received directly from commercial interest or their agents (eg, speakers' bureaus) from 10.13039/100004337Roche, 10.13039/100008067Genomic Health, and 10.13039/100004319Pfizer.

**Bethany Sleckman** has no conflict of interest.

**Reshma Mahtani** reports consulting fees from Agendia, Amgen, Biotheranostics, Daiichi-Sankyo, Eli Lilly, Genentech, Immunomedics, Merck, Pfizer, Novartis, SeaGen, Genentech, AstraZeneca, and Puma.

**Véronique Diéras** reports consulting fees from Genentech, Eli Lilly, Pfizer, AbbVie, Novartis Pharma KK, Roche-Peru, AstraZeneca, Daiichi, and reports fees for non-CME services received directly from commercial interest or their agents (eg, speakers' bureaus) from Pfizer, Novartis Pharma KK, Roche-Peru, and AstraZeneca.

## References

[1] Asghar U., Witkiewicz A.K., Turner N.C., Knudsen E.S. (2015). The history and future of targeting cyclin-dependent kinases in cancer therapy. Nat Rev Drug Discov.

[2] Finn R.S., Dering J., Conklin D., Kalous O., Cohen D.J., Desai A.J. (2009). PD 0332991, a selective cyclin D kinase 4/6 inhibitor, preferentially inhibits proliferation of luminal estrogen receptor-positive human breast cancer cell lines in vitro. Breast Cancer Res.

[3] Musgrove E.A., Caldon C.E., Barraclough J., Stone A., Sutherland R.L. (2011). Cyclin D as a therapeutic target in cancer. Nat Rev Cancer.

[4] Toogood P.L., Harvey P.J., Repine J.T., Sheehan D.J., VanderWel S.N., Zhou H. (2005). Discovery of a potent and selective inhibitor of cyclin-dependent kinase 4/6. J Med Chem.

[5] Turner N.C., Ro J., Andre F., Loi S., Verma S., Iwata H. (2015). Palbociclib in hormone-receptor-positive advanced breast cancer. N Engl J Med.

[6] National Comprehensive Cancer Network (2021). NCCN Clinical Practice Guidelines in Oncology (NCCN Guidelines®). Breast cancer. Version 5. National Comprehensive Cancer Network.

[7] Gennari A., Andre F., Barrios C.H., Cortes J., de Azambuja E., DeMichele A. (2021). ESMO Clinical Practice Guideline for the diagnosis, staging and treatment of patients with metastatic breast cancer. Ann Oncol.

[8] Finn R.S., Martin M., Rugo H.S., Jones S., Im S.A., Gelmon K. (2016). Palbociclib and letrozole in advanced breast cancer. N Engl J Med.

[9] Rugo H.S., Finn R.S., Dieras V., Ettl J., Lipatov O., Joy A.A. (2019). Palbociclib plus letrozole as first-line therapy in estrogen receptor-positive/human epidermal growth factor receptor 2-negative advanced breast cancer with extended follow-up. Breast Cancer Res Treat.

[10] Turner N.C., Slamon D.J., Ro J., Bondarenko I., Im S.A., Masuda N. (2018). Overall survival with palbociclib and fulvestrant in advanced breast cancer. N Engl J Med.

[11] Turner N.C., Finn R.S., Martin M., Im S.A., DeMichele A., Ettl J. (2018). Clinical considerations of the role of palbociclib in the management of advanced breast cancer patients with and without visceral metastases. Ann Oncol.

[12] Rugo H.S., Cristofanilli M., Loibl S., Harbeck N., DeMichele A., Iwata H. (2021). Prognostic factors for overall survival in patients with hormone receptor-positive advanced breast cancer: analyses from PALOMA-3. Oncologist.

[13] Cristofanilli M., Rugo H.S., Im S.A., Slamon D.J., Harbeck N., Bondarenko I. (2022). Overall survival with palbociclib and fulvestrant in women with HR+/HER2- ABC: updated exploratory analyses of PALOMA-3, a double-blind, phase III randomized study. Clin Cancer Res.

[14] Johnston S., O'Shaughnessy J., Martin M., Huober J., Toi M., Sohn J. (2021). Abemaciclib as initial therapy for advanced breast cancer: MONARCH 3 updated results in prognostic subgroups. NPJ Breast Cancer.

[15] Hortobagyi G.N., Stemmer S.M., Burris H.A., Yap Y.S., Sonke G.S., Hart L. (2021). Overall survival (OS) results from the phase III MONALEESA-2 (ML-2) trial of postmenopausal patients (pts) with hormone receptor positive/human epidermal growth factor receptor 2 negative (HR+/HER2−) advanced breast cancer (ABC) treated with endocrine therapy (ET) ± ribociclib (RIB) [abstract]. Ann Oncol.

