# Supplementary material for: Effect of palbociclib plus endocrine therapy on time to chemotherapy across subgroups of patients with hormone receptor‒positive/human epidermal growth factor receptor 2‒negative advanced breast cancer: Post hoc analyses from PALOMA-2 and PALOMA-3
Source: Breast. 2022 Nov 15;66:324–31. doi: 10.1016/j.breast.2022.11.005 (PMC9720565; doi:10.1016/j.breast.2022.11.005)
Supplement: Multimedia component 1 [file mmc1.docx]

# Supplemental Tables

## Supplemental Table 1. Follow-Up Systemic Anti-Cancer Therapy in PALOMA-2 and PALOMA-3 by Treatment Arm in the ITT population

| **Follow-up therapy, n (%)** | **PALOMA-2** | | **PALOMA-3** | |
| --- | --- | --- | --- | --- |
|  | **PAL+LET (n=444)** | **PBO+LET (n=222)** | **PAL+FUL (n=347)** | **PBO+FUL (n=174)** |
| No | 201 (45.3) | 61 (27.5) | 80 (23.1) | 30 (17.2) |
|  |  |  |  |  |
| Yes | 243 (54.7) | 161 (72.5) | 267 (76.9) | 144 (82.8) |
| Number of regimens |  |  |  |  |
| 1 | 93 (20.9) | 60 (27.0) | 65 (24.3) | 28 (19.4) |
| 2 | 66 (14.9) | 46 (20.7) | 58 (21.7) | 28 (19.4) |
| ≥3 | 84 (18.9) | 54 (24.3) | 144 (53.9) | 88 (61.1) |
| Not reported | 0 | 1 (0.5) | 0 | 0 |

## Supplemental Table 2. Commonly Used Subsequent Systemic Anti-Cancer Therapy (≥5% in any group) in PALOMA-2 by

## Treatment Arm

| **Therapy, n (%)** | **PAL+LET (n=444)** | **PBO+LET (n=222)** | **PAL+LET (n=444)** | **PBO+LET (n=222)** |
| --- | --- | --- | --- | --- |
|  | **First subsequent therapy** | | **Second subsequent therapy** | |
|  | **227 (51.1)** | **150 (67.6)** | **138 (31.1)** | **97 (43.7)** |
| Antihormonal | 138 (60.8) | 87 (58.0) | 50 (36.2) | 47 (48.5) |
| Fulvestrant | 70 (30.8) | 44 (29.3) | 23 (16.7) | 17 (17.5) |
| Exemestane | 49 (21.6) | 35 (23.3) | 19 (13.8) | 18 (18.6) |
| Tamoxifen | 16 (7.0) | 4 (2.6) | 5 (3.6) | 6 (6.2) |
| CDK 4/6 inhibitor |  |  |  |  |
| Palbociclib | 0 | 13 (8.7) | 1 (0.7) | 10 (10.3) |
| Chemotherapy | 83 (36.6) | 51 (34.0) | 87 (63.0) | 47 (48.5) |
| Paclitaxel | 29 (12.8) | 19 (12.7) | 19 (13.8) | 12 (12.4) |
| Capecitabine | 28 (12.3) | 19 (12.7) | 32 (23.2) | 25 (25.8) |
| Doxorubicin | 10 (4.4) | 6 (4.0) | 10 (7.2) | 2 (2.0) |
| Cyclophosphamide | 7 (3.1) | 7 (4.7) | 9 (6.5) | 3 (3.1) |
| Investigational drug | 23 (10.1) | 17 (11.3) | 7 (5.1) | 5 (5.2) |
| mTOR kinase inhibitor (everolimus) | 31 (13.7) | 26 (17.3) | 15 (10.9) | 7 (7.2) |

LET=letrozole; PAL=palbociclib; PBO=placebo.

## Supplemental Table 3. Commonly Used Subsequent Systemic Anti-Cancer Therapy (≥5% in any group) in PALOMA-3 by

## Treatment Arm

| **Therapy, n (%)** | **PAL+FUL (n=347)** | **PBO+FUL (n=174)** | **PAL+FUL (n=347)** | **PBO+FUL (n=174)** | **PAL+FUL (n=347)** | **PBO+FUL (n=174)** |
| --- | --- | --- | --- | --- | --- | --- |
|  | **First subsequent therapy** | | **Second subsequent therapy** | | **Third subsequent therapy** | |
|  | **267 (76.9)** | **144 (82.8)** | **204 (58.8)** | **121 (69.5)** | **153 (44.1)** | **92 (52.9)** |
| Angiogenesis inhibitor | 8 (3.0) | 3 (2.1) | 6 (2.9) | 1 (0.8) | 9 (5.9) | 3 (3.3) |
| Bevacizumab | 6 (2.2) | 3 (2.1) | 6 (2.9) | 1 (0.8) | 8 (5.2) | 2 (2.2) |
| Antihormonal | 114 (42.7) | 55 (38.2) | 41 (20.1) | 33 (27.3) | 53 (34.6) | 36 (39.1) |
| Exemestane | 64 (24.0) | 26 (18.1) | 21 (10.3) | 17 (14.0) | 27 (17.6) | 14 (15.2) |
| Fulvestrant | 19 (7.1) | 10 (6.9) | 3 (1.5) | 2 (1.7) | 8 (5.2) | 8 (8.7) |
| Letrozole | 13 (4.9) | 11 (7.6) | 6 (2.9) | 8 (6.6) | 14 (9.2) | 9 (9.8) |
| Tamoxifen | 12 (4.5) | 6 (4.2) | 9 (4.4) | 4 (3.3) | 9 (5.9) | 8 (8.7) |
| CDK 4/6 inhibitor | 8 (3.0) | 10 (6.9) | 5 (2.5) | 7 (5.8) | 8 (5.2) | 19 (20.7) |
| Palbociclib | 6 (2.2) | 8 (5.6) | 5 (2.5) | 7 (5.8) | 6 (3.9) | 14 (15.2) |
| Abemaciclib | 1 (0.4) | 0 | 0 | 0 | 2 (1.3) | 5 (5.4) |
| Chemotherapy | 143 (53.6) | 88 (61.1) | 147 (72.1) | 80 (66.1) | 138 (90.2) | 85 (92.4) |
| Capecitabine | 68 (25.5) | 36 (25.0) | 52 (25.5) | 22 (18.2) | 48 (31.4) | 29 (31.5) |
| Paclitaxel | 32 (12.0) | 32 (22.2) | 41 (20.1) | 18 (14.9) | 54 (35.3) | 37 (40.2) |
| Docetaxel | 14 (5.2) | 3 (2.1) | 2 (1.0) | 3 (2.5) | 4 (2.6) | 4 (4.3) |
| Cyclophosphamide | 13 (4.9) | 8 (5.6) | 9 (4.4) | 4 (3.3) | 29 (19.0) | 12 (13.0) |
| Doxorubicin | 12 (4.5) | 1 (0.7) | 8 (3.9) | 11 (9.1) | 42 (27.5) | 18 (19.6) |
| Gemcitabine | 7 (2.6) | 5 (3.5) | 6 (2.9) | 9 (7.4) | 34 (22.2) | 21 (22.8) |
| Eribulin | 7 (2.6) | 3 (2.1) | 13 (6.4) | 11 (9.1) | 56 (36.6) | 32 (34.8) |
| Vinorelbine | 6 (2.2) | 7 (4.9) | 10 (4.9) | 6 (5.0) | 34 (22.2) | 27 (29.3) |
| Carboplatin | 5 (1.9) | 1 (0.7) | 6 (2.9) | 5 (4.1) | 25 (16.3) | 10 (10.9) |
| Epirubicin | 3 (1.1) | 3 (2.1) | 2 (1.0) | 0 | 8 (5.2) | 7 (7.6) |
| Fluorouracil | 3 (1.1) | 2 (1.4) | 3 (1.5) | 1 (0.8) | 10 (6.5) | 10 (10.9) |
| Cisplatin | 2 (0.7) | 2 (1.4) | 2 (1.0) | 2 (1.7) | 9 (5.9) | 7 (7.6) |
| Methotrexate | 2 (0.7) | 3 (2.1) | 4 (2.0) | 2 (1.7) | 11 (7.2) | 8 (8.7) |
| Investigational drug | 12 (4.5) | 4 (2.8) | 8 (3.9) | 2 (1.7) | 13 (8.5) | 4 (4.3) |
| mTOR kinase inhibitor | 46 (17.2) | 22 (15.3) | 21 (10.3) | 15 (12.4) | 26 (17.0) | 13 (14.1) |
| Everolimus | 46 (17.2) | 22 (15.3) | 21 (10.3) | 15 (12.4) | 25 (16.3) | 13 (14.1) |

FUL=fulvestrant; PAL=palbociclib; PBO=placebo.
